# Reaching for Individualized Medicine in Diabetic Kidney Disease

**DOI:** 10.1001/jamanetworkopen.2025.1702

**Published:** 2025-03-03

**Authors:** Mallory Mandel, Sylvia E. Rosas

**Affiliations:** **Author Affiliations:** Division of Nephrology, Boston Children’s Hospital, Boston, Massachusetts (Mandel); Harvard Medical School, Boston, Massachusetts (Mandel, Rosas); Kidney and Hypertension Center, Joslin Diabetes Center, Boston, Massachusetts (Rosas).

In the US, an estimated 35.5 million people have chronic kidney disease (CKD), with 9 in 10 people unaware of the diagnosis.^1^ Albuminuria is both a marker of kidney disease and an independent risk factor for disease progression. Renin-angiotensin blockade with angiotensin-converting enzyme inhibitors or angiotensin-receptor blockers is the first-line standard of care for diabetic kidney disease therapy. Sodium-glucose cotransporter 2 (SGLT2) inhibitors can slow the progression of kidney disease and cardiovascular disease.^2^ The 2025 American Diabetes Association Standards of Care in Diabetes recommends that SGLT2 inhibitors be used in all patients with CKD (estimated glomerular filtration rate ≥20 mL/min/1.73 m^2^) and type 2 diabetes regardless of the presence of albuminuria.

Beernink and colleagues^3^ tested whether the effect of the SGLT2 inhibitor dapagliflozin on albuminuria would be similar on re-exposure as well as the feasibility of remote clinical assessment in a research trial. Twenty adults with type 2 diabetes with moderate albuminuria were included in this randomized, double-blind, placebo-controlled crossover trial using an n-of-1 approach. Participants collected first-morning urine samples using a home device and sent them to a central laboratory for measurement of urinary albumin-to-creatinine ratio (UACR). Wearable and digital devices were used to collect vital sign measurements, such as blood pressure and body weight. Capillary blood samples were also collected at home to assess clinical values, such as creatinine and plasma glucose levels, and were compared with a venous blood sample. Overall, dapagliflozin reduced albuminuria by a mean of −15.1% (95% CI −28.2% to −3.3%; *P* = .01) compared with placebo, with marked variability in individuals’ responses. Large ranges of UACR were found in both the dapagliflozin and the placebo periods; however, a significant correlation in individual UACR changes only occurred between dapagliflozin treatment periods (*r* = 0.50; *P* = .03). Reliable assessment of the UACR required assessing the mean of multiple samples, highlighting the importance of repeat measurement when assessing disease progression or treatment efficacy.

A major strength of the study by Beernink and colleagues^3^ is the n-of-1 crossover study design. Evidence-based medicine suggests that n-of-1 randomized clinical trials provide the strongest evidence of a treatment effect for an individual.^4^ Similar trial designs have been used in other settings, including hypertension trials, in the past.^5^ In contrast to population-level studies in which changes in albuminuria may be impacted by random day-to-day variation, the re-exposure design used in this study demonstrated a true pharmacological response at the individual level. Future research efforts are needed to determine the cause of the individual response variability. There are now several first-line therapies that delay progression of kidney disease in the context of type 2 diabetes, including SGLT2 inhibitors, nonsteroidal mineralocorticoid receptor antagonists, and glucagon-like peptide-1 receptor agonists. Each clinician would like to know which initial therapy would have the highest efficacy for the individual patient. While in the future, biomarkers may be identified to better guide decision-making, at this time, clinicians are using trial and error or a cumulative medication approach.

Advances in technology have paved the way for more accessible home screening devices and clinical tools. Beernink and colleagues^3^ used a urine home collection device, a validated tool for collecting and preserving small amounts of urine that was originally created in the Netherlands to collect urine samples from children. It consists of a small absorption pad, holder, and sample tube that can be refrigerated for days before analysis at a central laboratory. The authors found that the device was easy to implement, with 811 of 816 anticipated urine samples (99.4%) returned to the laboratory. In addition, it was easy for participants to measure blood pressure and body weight remotely. Remote capillary blood sample collection was rated as more challenging for participants to perform, although it was found to correlate significantly with venous blood creatinine measurements (*r* = 0.94; *P* < .001), suggesting feasibility of remote blood sample obtainment as well.

There is a growing trend toward personalized approaches to CKD monitoring and management. N-of-1 study designs and remote collection and monitoring could play important roles. Remote collection can facilitate both individual monitoring and large-scale decentralized clinical trials, with improved flexibility, reduced in-person visits, and decreased costs. On the individual level, response to treatment can be tracked in real time. Remote sample obtainment can also lower barriers for trial participants, which could lead to more representative cohorts, with increased inclusion of participants from resource-scarce areas, with less flexible work schedules, or facing transportation issues. Despite this excitement, n-of-1 studies and remote collection are not without limitations. They can be time intensive, as evidenced by the 16-month recruitment and study period despite the small sample size (20 patients) in this study. The study has limited generalizability, as all participants were White and most were men. While level of education is not reported, remote collection also requires a certain level of health literacy to operate the remote devices.

Screening for CKD in early stages is vital to initiate therapy. Individuals at risk for CKD, including patients with diabetes, hypertension, older age (>60 years), or family history of kidney disease, should be screened for glomerular filtration rate and albuminuria annually. In 2022, the US Food and Drug Administration granted approval for the first smartphone-powered home UACR test. Both the mailed urine collection device and the smartphone app method were compared in the Toward Home-Based Albuminuria Screening (THOMAS) study, a prospective, randomized implementation study including 15 000 Dutch participants between 2019 and 2021. The participation rate for the mailed urine method was higher compared with the smartphone method (59.4% vs 44.3%), with higher specificity (97.3% vs 67.9%).^6^ Participation for the smartphone app was lower for older individuals and participants living in a low socioeconomic status area, which raises important questions surrounding technology literacy, access, and affordability. The smartphone approach, however, eliminates the need for a centralized laboratory, and the generated results can be easily shared with a practitioner. Smartphone UACR testing technology is under active development and is improving.^7^ Policy-level interventions focused on increasing access and awareness of these devices will be key, as screening for at-risk individuals remains low.

Diabetic kidney disease management is undergoing an exciting transformation, and the field is moving toward prevention. Given the new medication classes, there is an urgent need for readily available and accessible CKD screening. Remote sample collection is feasible, offering an intriguing solution for both clinical and research practice. Beernink et al^3^ demonstrated that individual responses to these agents were highly heterogeneous. Thus, patients may benefit from a therapeutic regimen carefully tailored to their unique physiology. We anticipate that remote collection and decentralized or hybrid research studies will play an increasingly important role in the study of novel medications, whether alone or in combination, at the individual or population level.
